# Loss of the Na^+^/K^+^ cation pump CATP-1 suppresses *nekl*-associated molting defects

**DOI:** 10.1093/g3journal/jkae244

**Published:** 2024-10-21

**Authors:** Shaonil Binti, Philip T Edeen, David S Fay

**Affiliations:** Department of Molecular Biology, College of Agriculture, Life Sciences and Natural Resources, University of Wyoming, Laramie, WY 82071, USA; Department of Molecular Biology, College of Agriculture, Life Sciences and Natural Resources, University of Wyoming, Laramie, WY 82071, USA; Department of Molecular Biology, College of Agriculture, Life Sciences and Natural Resources, University of Wyoming, Laramie, WY 82071, USA

**Keywords:** *C. elegans*, molting, ion channels, catp-1, NimA kinases

## Abstract

The conserved *Caenorhabditis elegans* protein kinases NEKL-2 and NEKL-3 regulate membrane trafficking and are required for larval molting. Through a forward genetic screen, we identified a mutation in *catp-1* as a suppressor of molting defects in synthetically lethal *nekl-2; nekl-3* double mutants. *catp-1* encodes a membrane-associated P4-type ATPase involved in Na^+^–K^+^ exchange. A previous study found that wild-type worms exposed to the nicotinic agonist dimethylphenylpiperazinium (DMPP) exhibited larval arrest and molting-associated defects, which were suppressed by inhibition of *catp-1*. By testing spectrum *catp-1* alleles, we found that resistance to DMPP toxicity and the suppression of *nekl* defects did not strongly correlate, suggesting key differences in the mechanism of *catp-1*-mediated suppression. Through whole-genome sequencing of additional *nekl-2; nekl-3* suppressor strains, we identified 2 additional coding-altering mutations in *catp-1*. However, neither mutation, when introduced into *nekl-2; nekl-3* mutants using CRISPR, was sufficient to elicit robust suppression of molting defects, suggesting the involvement of other loci. Endogenously tagged CATP-1 was primarily expressed in epidermal cells within punctate structures located near the apical plasma membrane, consistent with a role in regulating cellular processes within the epidermis. Based on previous studies, we tested the hypothesis that *catp-1* inhibition induces entry into the predauer L2d stage, potentially accounting for the ability of *catp-1* mutants to suppress *nekl* molting defects. However, we found no evidence that loss of *catp-1* leads to entry into L2d. As such, loss of *catp-1* may suppress *nekl-*associated and DMPP-induced defects by altering electrochemical gradients within membrane-bound compartments.

## Introduction

Ecdysozoa, animals that undergo molting, encompass the major phyla Nematoda (roundworms) and Arthropoda (insects, chelicerates, crustaceans, and myriapods), as well as several smaller phyla including Tardigrada (tardigrades) and Nematomorpha (parasitic horsehair worms) (Aguinaldo *et al*. 1997; Ewer 2005; Telford *et al*. 2008). Common to ecdysozoans is a multilayered exoskeleton, the cuticle, which is an apical extracellular matrix secreted primarily by the epidermis (Sundaram and Pujol 2024). The material composition and mechanical properties of the cuticle can vary widely among different species and life stages. Whereas the cuticle is essential for movement, protection, and other basic life functions, it can pose limitations on developmental processes including animal growth. To accommodate growth and morphological changes, ecdysozoans undergo cycles of molting (ecdysis), whereby a new cuticle is synthesized underneath the old cuticle, which is shed. In the case of the nematode *Caenorhabditis elegans*, the shedding process occurs at the termination of each larval stage (L1, L2, L3, and L4), with the new cuticle allowing for body size expansion when resources are replete, or long-term survival (as dauer larvae; L3d) when conditions are deemed adverse (Singh and Sulston 1978; Fielenbach and Antebi 2008; Lazetic and Fay 2017b).

Progression through molting requires the coordination of several cellular processes including the ordered transcription of hundreds of molting-associated genes; the secretion and construction of a new cuticle; and the detachment, degradation, and partial recycling of the old cuticle and precuticle structures (Johnstone and Barry 1996; Page and Johnstone 2007; Lazetic and Fay 2017b; Miao *et al*. 2020; Tsiairis and Grosshans 2021; Sundaram and Pujol 2024). Correspondingly, mutations that disrupt these processes within the epidermal cells that underlie and secrete the cuticle can lead to defective molting and larval arrest. These include mutations affecting several steroid hormone receptors required for the cyclic transcription of molting genes, mutations in individual cuticle components and apical extracellular matrix regulators, and mutations that affect membrane trafficking including endocytosis and exocytosis (Asahina *et al*. 2000; Gissendanner and Sluder 2000; Kostrouchova *et al*. 2001; Frand *et al*. 2005; Monsalve and Frand 2012; Lazetic and Fay 2017b; Johnson *et al*. 2023). In addition, loss of individual plasma membrane proteins can lead to molting arrest. One example is LRP-1/megalin, an epidermal apolipoprotein receptor required for the internalization of environmental sterols via clathrin-mediated endocytosis (Yochem *et al*. 1999; Kang *et al*. 2013). Because cholesterol uptake is essential for the synthesis of steroid hormones in *C. elegans*, a failure to internalize LRP-1 may preclude the normal transcriptional activation of molting genes (Merris *et al*. 2003; Entchev and Kurzchalia 2005; Martin *et al*. 2010; Lazetic and Fay 2017b; Joseph *et al*. 2020; Binti *et al*. 2022).

Through genetic screens, we previously identified 2 *C. elegans* NimA-related kinases, NEKL-2 and NEKL-3, as essential for the completion of molting (Yochem *et al.* 2015). Specifically, null alleles of *nekl-2* or *nekl-3* lead to penetrant molting defects and larval arrest at the L1/L2 transition, whereas partial reduction-of-function alleles cause larvae to arrest at L2/L3. NEKL-2 is most similar to human NEK8 and NEK9, whereas NEKL-3 is orthologous to NEK6 and NEK7. Consistent with these findings, our genetic screens identified conserved ankyrin repeat partners of NEKL-2 (MLT-2/ANKS6 and MLT-4/INVS) and NEKL-3 (MLT-3/ANKS3) as required for molting, which we showed are necessary for proper NEKL subcellular localization (Lazetic and Fay 2017a). Further studies have indicated roles for NEKL-2 and NEKL-3 at multiple steps of membrane trafficking, including clathrin-mediated endocytosis and endosomal transport, and have implicated human NEK6 and NEK7 in analogous trafficking functions in tissue culture cells (Joseph *et al*. 2020, 2023).

In previous work, we described a genetic screen to identify suppressors of molting defects in *nekl* mutants (Joseph *et al*. 2018). Specifically, we combined weak aphenotypic reduction-of-function alleles of *nekl-2* and *nekl-3* to create synthetically lethal *nekl-2(fd81); nekl-3(gk894345)* double mutants, which were used to identify suppressors of *nekl* synthetic lethality. Our screen led to the identification of mutations affecting several core components of membrane trafficking as well as ECM modifiers and cell signaling components (Joseph *et al*. 2018, 2022; Lazetic *et al*. 2018). In addition, our studies demonstrated that mutations and environmental conditions that promote induction of the dauer pathway can suppress a subset of *nekl* alleles that arrest at the L2/L3 transition (Binti *et al*. 2022). Namely, entry into the uncommitted predauer state, L2d, can be sufficient to suppress molting defects and to partially restore the expression of molting-associated genes.

Here, we report a novel *nekl* suppressor mutation affecting CATP-1, a P4-type Na^+^–K^+^ pump. CATP-1 functions to maintain an electrochemical gradient across membranes by exporting 3 Na^+^ ions and importing 2 K^+^ ions during each ATP-driven catalytic cycle (Kaplan 2002; Morth *et al*. 2011; Clausen *et al*. 2017). Loss of *catp-1* suppresses larval arrest caused by exposure of worms to dimethylphenylpiperazinium (DMPP) (Ruaud and Bessereau 2007), a nicotinic agonist that uncouples the coordinated timing of cell divisions with molting cycles (Ruaud and Bessereau 2006). This study also provided evidence that CATP-1 is expressed and functions in the epidermis, consistent with a possible role in molting. In addition, evidence suggested that CATP-1 may be an effector of the dauer pathway, potentially linking to our finding that dauer induction can partially bypass the requirement for NEKL kinases (Binti *et al*. 2022). More recently, CATP-1 was shown to function in glial cells that associate with mechanosensory neurons involved in touch sensitivity (Johnson *et al*. 2020). Our current studies demonstrate that loss of CATP-1 ion pump activity can suppress molting defects in *nekl-2(fd81); nekl-3(gk894345)* mutants. In contrast to expectations, however, our data do not support the model that *catp-1*-mediated suppression occurs through induction of the dauer pathway, raising the possibility that changes in ion balance may impact molting through other mechanisms including possible effects on membrane trafficking.

## Materials and methods

### Strains, maintenance, mutagenesis

*C. elegans* strains were cultured on nematode growth medium (NGM) spotted with *E. coli*OP50 and were maintained at 22°C (unless otherwise stated) according to standard protocols (Stiernagle 2006). All strains used in this study are listed in Supplementary File 1. Mutagenesis was carried out using ethyl methanesulfonate (*fd168*, *fd170*) or *N*-ethyl-*N*-nitrosourea (ENU; *fd274*) as previously described (Kutscher and Shaham 2014; Joseph *et al*. 2018).

### Genome sequencing

Whole-genome sequencing (WGS) of strains WY1286 (5 × backcross), WY1744 (3 × backcross), and WY1768 (1 × backcross) was carried out as described (Joseph *et al*. 2018). After sequencing of WY1286, the sibling subtraction method (Joseph *et al*. 2018) was carried out to reduce the number of candidate causal mutations.

### CRISPR/Cas9 genome editing

Standard methods were used to design and generate desired genomic changes (Arribere *et al*. 2014; Kim *et al*. 2014; Paix *et al*. 2014, 2015; Fay *et al*. 2021; Ghanta *et al*. 2021). Oligos including sgRNAs, repair templates, and amplification primers used in this study are listed in Supplementary File 2. CATP-1–fluorescent protein fusion reporters were generated using methods from Dickinson *et al*. (2015) and Dickinson and Goldstein (2016). All CRISPR-generated mutations were also confirmed by sequencing.

### *catp-1(ok2585)* haploinsufficiency assay

Tests for dominance/haploinsufficiency were carried out as previously described (Joseph *et al*. 2018). Briefly, *nekl-2(fd81); nekl-3(gk894345); fdEx286* males were crossed to *nekl-2(fd81) catp-1(ok2585); nekl-3(gk894345); fdEx286* hermaphrodites and cross-progeny males were scored for the presence of the *nekl-3*^+^*GFP^+^* rescuing extrachromosomal array (*fdEx286*). Haploinsufficiency would be expected to result in suppressed *nekl-2(fd81) catp-1(ok2585)/+; nekl-3(gk894345)* adult males that do not require the array. Of the 101 adult males cross-progeny scored, 101 contained *fdEx286*, strongly arguing against suppression by *ok2585* haploinsufficiency.

### DMPP treatment and arrest assay

A 75-mM stock solution of DMPP was prepared by adding 0.239 gof DMPP to 10 ml of (reverse-osmosis) water and heating to 50°C to solubilize. DMPP plates were prepared by adding 2 ml of DMPP to 198 ml of autoclaved NGM solution for a final DMPP concentration of 0.75 mM. 5 ml was poured into individual 35-mm plates, allowed to solidify, and spotted with 250 µl of an overnight OP50 culture. Ten young adult worms were placed on each plate (2 plates per strain) and allowed to lay eggs for 8 h. Counts were made of viable/arrested worms after ∼3 days.

### Image acquisition and analysis

Confocal fluorescence images were acquired using an Olympus IX83 inverted microscope with a Yokogawa spinning-disc confocal head (CSU-W1). z-Stack images with a step size of 0.2 µm were acquired using a 100×, 1.35 N.A. silicone oil objective. cellSense 3.1 software (Olympus Corporation) was used for image acquisition. DIC images and fluorescence images were obtained using a Nikon Eclipse epifluorescence microscope using 10×, 0.25 N.A. and 40×, 0.75 N.A. objectives. Image acquisition was controlled by Openlab 5.0.2 software (Agilent Inc.). Before they were imaged, animals were mounted on 3% agarose pads and anesthetized using 1 mM of levamisole in M9 buffer. Image processing and analysis were carried out using FIJI software (Schindelin *et al*. 2012).

### Synchronization and analysis of molting

Assays for molting arrest and genetic suppression were carried out as described (Lazetic and Fay 2017a; Joseph *et al*. 2018). To generate synchronized populations of L1 larvae, gravid adults were subjected to treatment with bleach, after which eggs were washed at least 5 times and allowed to hatch in M9 buffer overnight at room temperature with gentle rotation (Porta-de-la-Riva *et al*. 2012). Hatched larvae were transferred to NGM/OP50 plates, and pharyngeal pumping or P*_mlt-10_*::GFP–PEST (Frand *et al*. 2005) expression was recorded every hour at 20°C using an Olympus MVX10 MacroView microscope equipped with a 2 × objective. Molting was defined as when ≤50% animals (*n* = 10) were pumping or when ≥50% expressed P*_mlt-10_*::GFP–PEST.

### Statistical analysis and data availability

All statistical tests were performed using GraphPad Prism 10 following established protocols (Fay and Gerow 2013).

## Results

### Loss of the cation exchanger CATP-1 suppresses molting defects in *nekl-2; nekl-3* mutants

**Fig. 1.**
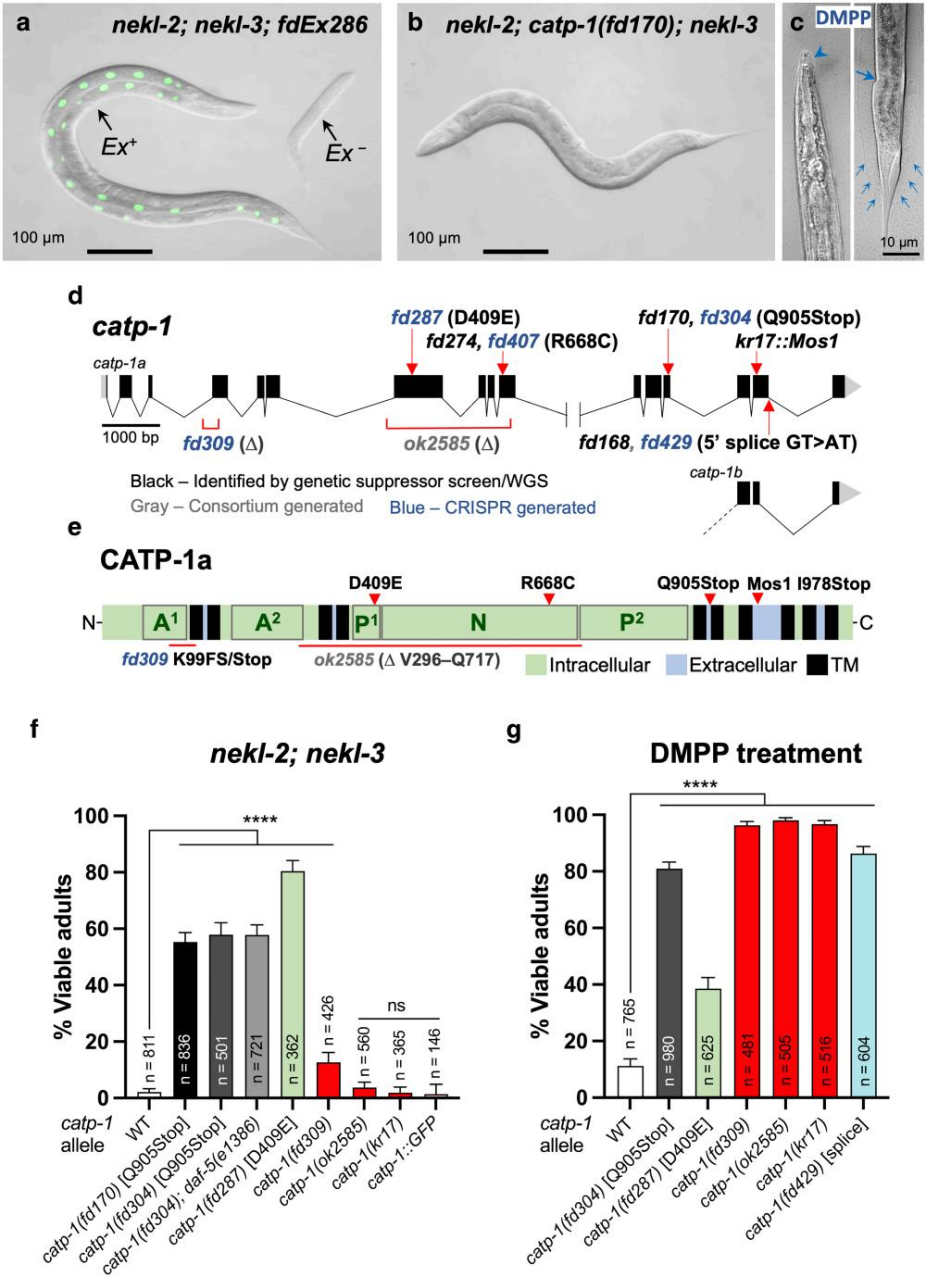
Mutations in *catp-1* lead to suppression of *nekl* molting and DMPP-induced arrest. DIC and GFP image overlays of a) *nekl-2(fd81); nekl-3(gk894345)* and b) *nekl-2(fd81); catp-1(fd170); nekl-3(gk894345)* strains. Whereas the GFP-positive adult in a carries the *fdEx286* extrachromosomal array (*Ex^+^*) containing rescuing wild-type *nekl-3* genomic sequences and the *sur-5*::GFP reporter, the nonrescued GFP-negative sibling larva (*Ex^−^*) is terminally arrested at the L2/L3 molt. In contrast, ∼50% of *nekl-2(fd81) catp-1(fd170); nekl-3(gk894345)* worms (b) do not exhibit molting defects and reach adulthood. c) Images of molting-defective wild-type (N2) worms on DMPP plates. Arrowhead in the left panel indicates cuticle covering the buccal cavity. Small arrows in the right panel indicate cuticular material surrounding the tail region; large arrow, constriction of the midbody, which is observed in other molting-defective larvae. d) Schematic of the *catp-1* genomic locus showing the locations of the relevant mutants generated for this study. Alternatively spliced forms of *catp-1a* and *catp-1b*, which differ at C-terminal exons 15 and 16 only, are indicated. e) Protein diagram of CATP-1a indicating the predicted locations of TM domains; intracellular and extracellular regions; and protein domains including actuator (A^1^, A^2^), phosphorylation (P^1^, P^2^), and ATP-binding (N) domains. The locations of several key amino acid variants are also indicated. f) Percentage of viable adults for the indicated genotypes, which include *nekl-2(fd81); nekl-3(gk894345)* mutations in addition to the indicated mutations in *catp-1*; WT indicates control strain containing a wild-type *catp-1* allele. g) Percentage of viable adults following DMPP treatment for the indicated genotypes. Error bars in e and f indicate 95% confidence intervals; *P*-values were calculated using Fisher’s exact text; ****P < 0.0001.

In a genetic screen for suppressors of *nekl-2(fd81); nekl-3(gk894345)* synthetic lethality, we isolated strain WY1286 [*nekl-2(fd81) fd170; nekl-3(gk894345)*] (Joseph *et al*. 2018). Whereas 2% of *nekl-2(fd81); nekl-3(gk894345)* (hereafter *nekl-2; nekl-3*) mutants developed into viable adults because of molting defects, > 50% of *nekl-2fd170; nekl-3* triple mutants progressed to adulthood within ∼3 or 4 days (Fig. 1a, b, and f). Backcrossing of WY1286 further suggested that genetic suppression was caused by a single-locus and autosomal recessive mutation. Consistent with this, WGS, in conjunction with the sibling subtraction method (Joseph *et al*. 2018), indicated that the suppressor mutation was on chromosome I and identified 3 point mutations predicted to alter protein coding sequences within the distal right arm of chromosome I. These included a G > A transition resulting in a premature Q905Stop in CATP-1 (CAG > TAG) (Fig. 1d; Supplementary Fig. 1), a C > T transition causing an A341T substitution in F22G12.4 (GCA > ACA), and a G > A transition leading to a G253E substitution in TAF-1 (GGA > GAA). Introduction of an equivalent *catp-1* Q905Stop mutation into *nekl-2; nekl-3* mutants using CRISPR/Cas9 methods (*fd304*) (Farboud and Meyer 2015; Farboud *et al*. 2019; Fay *et al*. 2021) led to adult viability of 58%, similar to that observed for *nekl-2catp-1(fd170);nekl-3* animals (Fig. 1d–f). These findings indicate that *catp-1(fd170)* is the causative suppressor mutation in strain WY1286. We note that *catp-1(RNAi)* failed to suppress molting defects in *nekl-2; nekl-3* mutants, possibly due to insufficient knockdown of CATP-1 by this method (also see below). In addition, we did not observe obvious morphological defects in *catp-1* single mutants, although *catp-1* strains took slightly longer to reach adulthood as compared to wild type (see below).

*catp-1* is predicted to encode a P4-type ATPase alpha subunit, which in conjunction with beta and gamma subunits forms a membrane-associated cation pump (Ruaud and Bessereau 2007). In *C. elegans*, CATP-1 is most similar to CATP-2 and CATP-3—both are 41% identical and 61% similar to CATP-1—and CATP-1 is 30% identical and 49% similar to CATP-4. With respect to human family members, CATP-1 is most similar to the alpha type IIC subfamily members ATP1A2 and ATP1A3, which function as ATP-dependent Na^+^/K^+^ exchangers (Kuhlbrandt 2004; Ruaud and Bessereau 2007; Farley 2012). Acting at the plasma membrane, members of this subfamily export 3 Na^+^ ions and internalize 2 K^+^ ions during each catalytic cycle, thereby producing an inside–outside electrochemical gradient. The predicted domain structure of CATP-1a includes 10 transmembrane (TM) domains, along with bipartite intracellular actuator (A1/A2) and phosphorylation (P1/P2) domains and a contiguous intracellular ATP-binding (N) domain (Fig. 1e; Supplementary Fig. 1). A second predicted isoform, CATP-1b, has an altered C terminus and contains sequences through the first 8 TM domains (Fig. 1d; Supplementary Fig. 1). The Q905Stop mutations [(*catp-1(fd170)* and *catp-1(fd304)*] are predicted to remove the C-terminal 216 and 140 amino acids of CATP-1a and CATP-1b isoforms, respectively (Fig. 1d; Supplementary Fig. 1).

To determine whether elimination of CATP-1 cation pump activity is relevant to its role in the context of *nekl* suppression, we used CRISPR/Cas9 to modify a highly conserved aspartic acid residue (D409) within the conserved D–K–S/T–G–T sequence of the P1 domain (Fig. 1d; Supplementary Fig. 1) (Ohtsubo *et al*. 1990; Ruaud and Bessereau 2007). During the cation exchange process, this aspartic acid is transiently phosphorylated, leading to the formation of a phosphoenzyme intermediate and a change in conformational state (Post and Kume 1973; Morth *et al*. 2011; Clausen *et al*. 2017). Notably, CATP-1 D409E robustly suppressed *nekl-2; nekl-3* synthetic lethality (∼80% viable; Fig. 1f), indicating that reduction or loss of the CATP-1 ion pump activity is likely critical to its mechanism of *nekl* suppression. The greater suppression level observed for CATP-1(D409E) relative to that induced by the Q905Stop mutation could be due to partial activity retained by of the truncated protein produced by the CATP-1(Q905Stop) mutation. Taken together, our results indicate that reduction or loss of CATP-1 cation exchange function leads to the suppression of *nekl-2; nekl-3* molting defects.

We next tested 2 previously isolated alleles of *catp-1* to determine whether they could suppress *nekl-2; nekl-3* lethality. *catp-1(kr17)* was originally identified as a suppressor of DMPP toxicity anWBVar02159856d contains a ∼1.5-kb *Mos1* transposon insertion in the 15th exon of *catp-1* (Fig. 1d). The insertion leads to a stop codon after I978, within an extracellular loop following the 7th TM domain of CATP-1 (Fig. 1e; Supplementary Fig. 1) (Ruaud and Bessereau 2007). We also tested a putative null deletion allele of *catp-1(ok2585)*, which contains a 2,132-bp deletion spanning intron 6 to exon 10 removing a majority of the catalytic domain (V296–Q717; Fig. 1d and e; Supplementary Fig. 1) (C. elegans Deletion Mutant Consortium 2012). Somewhat surprisingly, neither *kr17* nor *ok2585* conferred suppression when introduced into the *nekl-2; nekl-3* background, indicating that suppression is dependent on the specific properties of individual *catp-1* alleles (Fig. 1f). Additionally, *catp-1(ok2585)* failed to suppress the single-mutant hypomorphic alleles *nekl-3(sv3)* and *mlt-4(sv9)* (Supplementary Fig. 2a). As a further test, we generated a CRISRP-based deletion allele (*fd309*) that removes portions of intron 3 and exon 4 (Fig. 1d and e; Supplementary Fig. 1). This deletion would be predicted to result in exon skipping and the termination of CATP-1 following position K99. Although *catp-1*(*fd309*) conferred weak suppression of *nekl-2; nekl-3* mutants, the lack of robust suppression observed for all 3 *catp-1* alleles suggests that strong reduction-of-function mutations, including nulls, may be relatively ineffective at suppressing *nekl* molting defects (Fig. 1f). One possible explanation for this observation is that strong loss of CATP-1 function may lead to the compensatory upregulation of *catp-2*, as has been reported for *catp-1(kr17)* (Johnson *et al*. 2020). At the same time, we failed to detect genetic suppression in *nekl-2catp-1(ok2585)/+; nekl-3* worms, in which *catp-1(ok2585)* was heterozygous, suggesting that a ∼50% reduction in CATP-1 activity (haploinsufficiency) is not sufficient for suppressing *nekl-2; nekl-3* molting defects (see Materials and methods).

### *catp-1* mutations identified in 2 other sequenced strains are insufficient for suppression

WGS of 2 other suppressor strains, WY1768 (*nekl-2; fd168; nekl-3*) and WY1744 (*nekl-2fd274; nekl-3*), identified 2 additional mutations predicted to alter CATP-1. Whereas strain WY1744 exhibited 34% adult viability, strain WY1768 was ∼85% viable, placing it among the strongest suppressors identified by our screen (Fig. 1c and d; Supplementary Fig. 2b) (Joseph *et al*. 2018). Both strains were sequenced after minimal backcrossing and without the sibling subtraction method. Whereas WGS of WY1744 identified 9 coding mutations, 7 of which were on chromosome I, sequencing of WY1768 identified 14 coding mutations, of which only 3 were on chromosome I (Supplementary Fig. 2c and d).

In the case of strain WY1744, the mutation in *catp-1* led to an R668C substitution in a residue directly adjacent to a highly conserved arginine required for ATPase activity (R669; Fig. 1c and d; Supplementary Fig. 1) (Jacobsen *et al*. 2002; Ruaud and Bessereau 2007; Morth *et al*. 2011; Clausen *et al*. 2017). Nevertheless, BLAST searches indicate that R668 is less highly conserved than R669 within nematodes; the equivalent position in CATP-1 in *Caenorhabditis bovis* and *Wuchereria bancrofti* is a cysteine and a lysine, respectively. The mutation in strain WY1768 alters the conserved 5′ splice site of *catp-1a* intron 15 (GT > AT; Fig. 1d). Failure to incorporate exon 16 would remove the 62 C-terminal amino acids of CATP-1a, including portions of the ninth TM domain, the 10th TM domain, and the C-terminal 21 amino acids (Fig. 1d; Supplementary Fig. 1). In contrast, the shorter CATP-1b isoform would not be expected to be altered by the splice-site mutation (Fig. 1d).

CRISPR methods were used to introduce analogous R668C (*fd407*) and *catp-1a* intron-15 splice-site (*fd429*) mutations into the wild-type *catp-1* locus in *nekl-2; nekl-3* mutants (Fig. 1d and e). Surprisingly, neither mutation conferred robust suppression, although *fd407* gave rise to ∼6% adult viability, which was slightly above background (*P* < 0.001; Supplementary Fig. 2b). These results demonstrate that neither mutation in *catp-1* is sufficient to suppress molting defects in *nekl-2; nekl-3* mutants and implicate other loci as at least partially required for the observed genetic suppression in these strains. To determine whether the detected mutations in *catp-1* might partially contribute to the suppression observed in WY1744 and WY1768 strains, we attempted to revert the *catp-1* mutations in these backgrounds using CRISPR methods but were unsuccessful for technical reasons. We also attempted transgenic rescue experiments using *catp-1a* cDNA under the control of a hypodermal-specific promoter (Y37A1B.5; P_hyp7_::CATP-1), including a construct that encodes an in-frame N-terminal GFP (P_hyp7_::GFP::CATP-1). Unfortunately, both *catp-1* expression constructs appeared to be highly toxic, even when diluted with co-injection markers. As such, we were unable to determine the extent to which these mutations in *catp-1* might contribute to the suppression observed these strains. Nevertheless, our findings highlight the importance of follow-up tests to determine the functional role of mutations discovered by WGS, including mutations in genes that have been shown to be causative based on other alleles.

### Suppression of DMPP toxicity by *catp-1* mutations

*catp-* 1 was originally identified as a suppressor of larval lethality caused by exposure to the nicotinic agonist DMPP. We were therefore curious to determine whether the *catp-1* alleles generated by our study would also suppress DMPP-induced lethality. To test this, we generated strains containing *catp-1(fd304)* [Q905Stop], *catp-1(fd309)* [K99FS], *catp-1(fd287)* [D409E], and *catp-1(fd429)* [GT > AT splice site] in wild-type backgrounds and assayed DMPP sensitivity along with *catp-1(kr17)*, *catp-1(ok2585*), and wild type. In wild-type worms, DMPP treatment resulted in a penetrant larval growth arrest and/or growth retardation, with many worms exhibiting molting defects (Fig. 1c). As expected, *catp-1(kr17)* exhibited robust suppression of DMPP lethality, as previously reported (Fig. 1g; Supplementary Fig. 2e) (Ruaud and Bessereau 2007). Notably, the putative null deletion allele, *catp-1(ok2585)*, conferred the highest level of viability on DMPP, despite failing to suppress *nekl*-associated molting defects (Fig. 1g; Supplementary Fig. 2e). Moreover, the splice-site mutation, *catp-1*(GT > AT) conferred moderate resistance to DMPP, indicating that loss of *catp-1a* coding sequences after exon 15 does impact the function of the *catp-1* locus even though this allele was insufficient at suppressing *nekl* molting defects (Fig. 1f and g; Supplementary Fig. 2e). Likewise, strong suppression was observed for several other alleles including *catp-1*(Q905Stop) and the N-terminal deletion, *catp-1(fd309)* (Fig. 1g). Curiously, although the cation pump impaired mutant, *catp-1*(D409E), was strongest at suppressing *nekl* defects, it appeared to be the weakest of the tested DMPP suppressors (Fig. 1g), consistent with previous findings that CATP-1 may have pump-independent functions (Ruaud and Bessereau 2007). Overall, our results suggest key differences in the mechanisms by which reduction of CATP-1 function leads to the suppression of *nekl-*associated molting defects vs DMPP resistance.

### CATP-1 is expressed in apical puncta in the epidermis

CATP-1 was previously shown to be expressed and to function in epidermal cells including the hypodermal syncytia in the head (hyp1 to hyp6), midbody (hyp7), and tail (hyp8 to hyp11) (Ruaud and Bessereau 2007). hyp7 is also the tissue focus for NEKL and MLT activities and is the major contributor to the larval and adult cuticles (Yochem *et al*. 2015; Lazetic and Fay 2017a, 2017b). CATP-1 was also reported to function in glial cells that surround touch-sensory neurons in the head (Johnson *et al*. 2020). In addition, modENCODE RNA sequencing data available on WormBase indicate that CATP-1 is expressed throughout development, including in embryos and all 4 larval stages, with highest levels corresponding to dauer larvae (Li *et al*. 2014; Harris *et al*. 2020; Sternberg *et al*. 2024).

**Fig. 2.**
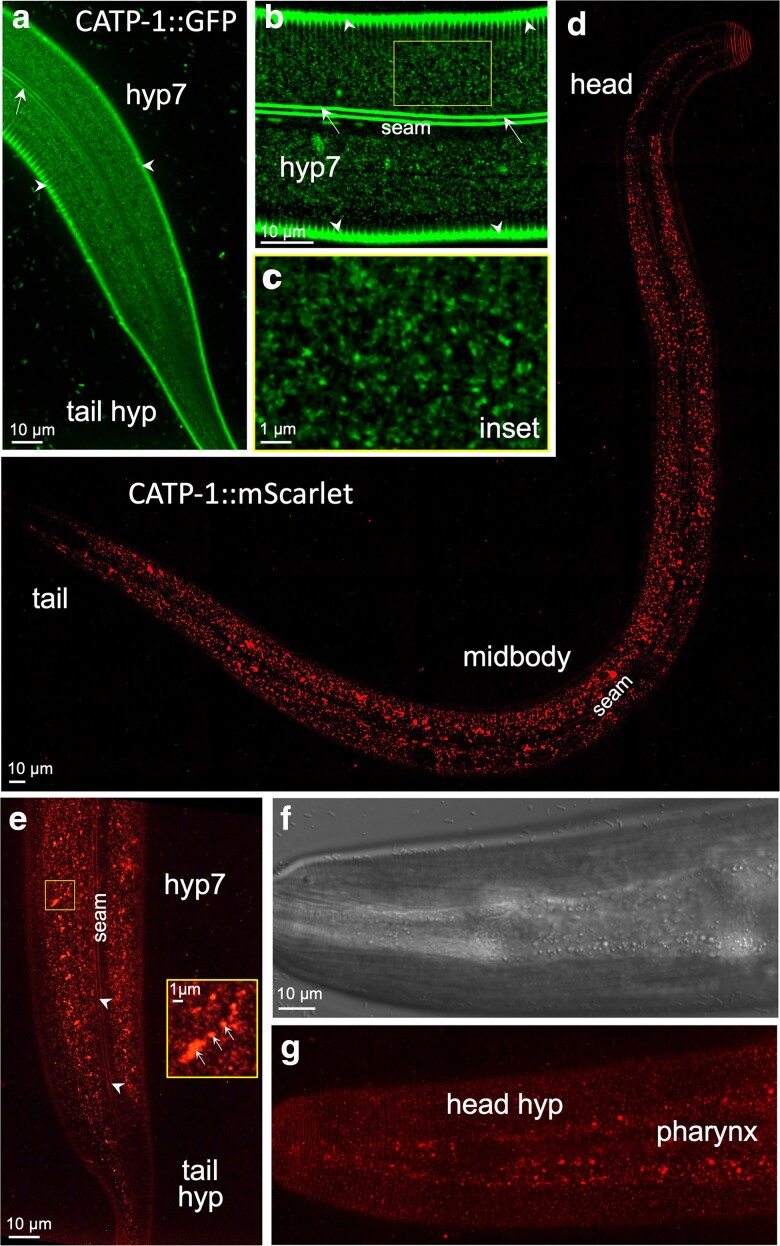
CATP-1 is expressed predominantly in punctate structures within the epidermis. Fluorescence (a–e, g) and DIC (f) images of worms expressing endogenously tagged CATP-1::GFP (a–c) and CATP-1::mScarlet (d–g), in which sequences encoding fluorescent proteins were integrated at the C terminus of the *catp-1a* isoform. All panels show young adults. a) Apical regions corresponding to the midbody (hyp7 syncytium) and tail (hyp8–11) are indicated. b) Midbody apical section of hyp7 and c) enlarged inset showing punctate and reticular structures. In a)–c), autofluorescence of the dorso-ventral cuticle (white arrowheads) and lateral cuticular alae (white arrows) is indicated, along with the location of the seam cell (b), in which CATP-1::GFP was not detected. d) Whole-body apical image of a young adult expressing CATP-1::mScarlet with relevant regions indicated. e) CATP-1::mScarlet expression in the apical midbody and tail regions along with enlarged inset. Note the presence of fluorescence signals in variably sized punctate structures including accumulation of mScarlet in larger structures that likely correspond to late endosomes and lysosomes (white arrows in inset). White arrowheads in e indicate autofluorescence of cuticular alae. f) DIC and g) corresponding fluorescence images of CATP-1::mScarlet in the apical head region showing expression in both the anterior hypodermis (hyp1–6) and pharynx, in which larger puncta accumulate.

To further examine the tissue expression and subcellular localization of CATP-1, we obtained endogenous CRISPR-tagged variants in which the CATP-1a isoform was tagged at its C terminus with either GFP or mScarlet. Consistent with previous studies, we observed clear expression of CATP-1::GFP and CATP-1::mScarlet in the hyp7 midbody, as well as in head and tail epidermal cells of young adults (Fig. 2a–g); expression at earlier larval stages was more difficult to analyze because of competing autofluorescence coming from the intestine. With respect to subcellular localization, we observed faint CATP-1::GFP in punctate and reticular structures in the apical region of hyp7 underlying the cuticle, which may correspond to membrane or juxtamembrane accumulations including clathrin-coated pits and/or recycling endosomes (Fig. 2a–c). Likewise, faint CATP-1::mScarlet fluorescence was observed in apical epidermal puncta but was also detected at higher levels in more medial compartments, which may correspond to late endosomes and lysosomes (Fig. 2d and e). This difference in GFP and mScarlet localization is likely explained by the observation that red fluorescent proteins can be cleaved from their translational fusion partners leading to their accumulation in acidic compartments of the endo-lysosome system in worms (Clancy *et al*. 2023). In addition, CATP-1::mScarlet was observed in punctate structures within the region of the pharynx (Fig. 2f and g). In contrast, both CATP-1::GFP and CATP-1::mScarlet were largely absent from epidermal seam cells (Fig. 2a, b, d, and e). The absence of apparent CATP-1 expression in nervous system–associated structures, such as head glia, may reflect sensitivity limitations of our reporters. Collectively, our data are consistent with CATP-1 playing a role in the epidermis during the molting process.

We note that tagging the C terminus of CATP-1b did appear to reduce its activity based on the DMPP resistance assay. Namely, both *catp-1::GFP* and *catp-1::mScarlet* alleles conferred resistance to DMPP-induced larval arrest, although the degree of suppression was less than that observed for the putative null allele, *catp-1(ok2585)* (Supplementary Fig. 1e). In contrast, *catp-1::GFP* failed to suppress *nekl-2: nekl-3* molting defects, indicating that the CATP-1::GFP fusion protein retains partial activity (Fig. 1f).

### CATP-1 loss does not appear to trigger entry into L2D

Suppression of DMPP toxicity was previously observed under environmental conditions that induce the formation of dauers and in genetic mutants that cause constitutive dauer entry (Ruaud and Bessereau 2006; Ruaud *et al*. 2011). Data from these studies further suggested that DMPP resistance may correlate with entry into L2d, a predauer stage from which worms can either transition into dauers (L3d) or resume continuous development by entering L3. Notably, the duration of L2d is typically ∼1.2–2-fold longer than that of L2, depending on the strength and nature of the inducing signal, and suppression of DMPP toxicity by *daf-2* mutants is positively correlated with the extent of L2 elongation (Golden and Riddle 1984; Ruaud *et al*. 2011; Karp 2018; Binti *et al*. 2022). Consistent with this, *catp-1(kr17)* was reported to extend the length of L2 by ∼1.6-fold but to have no effect on the duration of L1 or other larval stages (Ruaud and Bessereau 2007).

Recently, we showed that induction of L2d is also sufficient to suppress molting defects in ∼50% of *nekl-2; nekl-3* mutants (Binti *et al*. 2022). We thus hypothesized that loss of *catp-1* may suppress *nekl-2; nekl-3* molting defects by inducing L2d. As a first test, we introduced a dauer-defective allele of *daf-5* (*e1386*) (Riddle *et al*. 1981) into *nekl-2catp-1; nekl-3* mutants to inhibit entry into L2d. Notably, we had previously shown that *daf-5(e1386)* significantly reduces the ability of dauer-inducing conditions to suppress *nekl-2; nekl-3* molting defects (Binti *et al*. 2022). However, we observed no difference in the level of suppression in *nekl-2catp-1; daf-5; nekl-3* mutants vs *nekl-2catp-1; nekl-3* mutants (Fig. 1e), suggesting that loss of *catp-1* does not suppress *nekl* molting defects through an L2d mechanism or that loss of *catp-1* induces L2d via a mechanism that is independent of DAF-5 function.

**Fig. 3.**
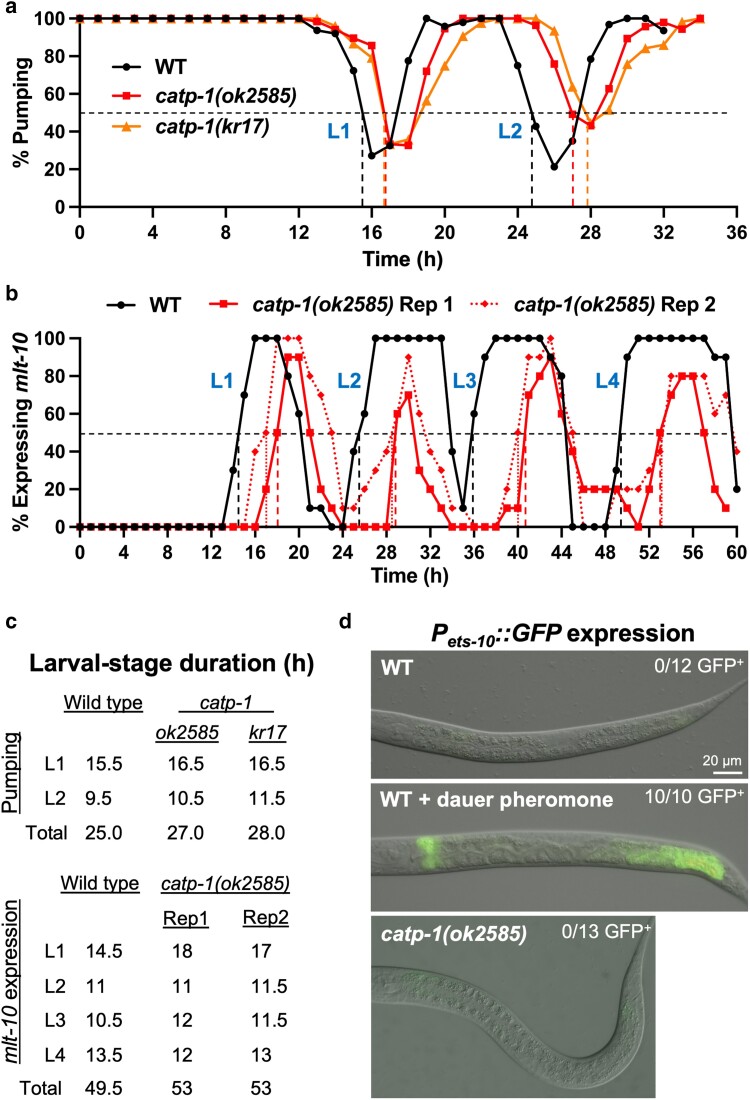
Loss of *catp-1* does not substantially extend the length of the L2 stage nor does it induce an L2d marker. a) Cessation of pharyngeal pumping was used to measure the duration of L1 and L2 stages in the indicated backgrounds. b) P*_mlt-10_*::GFP–PEST expression was used to measure the lengths of larval stages in wild-type and *catp-1* larvae; 2 replicates are shown for *catp-1*. Dashed vertical lines in a and b indicate the point at which ≥50% of larvae had entered lethargus for a given stage. c) Quantification of larval-stage lengths for a and b. d) Expression of P*_mlt-10_*::GFP–PEST in L2 larvae under the indicated conditions. In contrast to wild type with dauer pheromone, neither untreated wild type nor *catp-1* mutants express the L2d marker. For b) and c), strains were grown at 20°C to match the temperatures used in studies by Ruaud and Bessereau (2007).

To further test for the induction of L2d in *catp-1* mutants, we examined the timing of larval molts in wild type and *catp-1* mutants using 2 methods. In one assay, we followed molts by scoring pharyngeal pumping in a synchronized population of worms released from early L1-larval arrest; loss of pumping occurs when animals enter the lethargus phase of the molting cycle and is commonly used to measure the duration of larval stages (Singh and Sulston 1978; Lazetic and Fay 2017b). For pumping studies, we used the previously characterized *catp-1*(*kr17*) allele as well as the putative null allele, *catp-1*(*ok2585*), which we found strongly suppressed DMPP toxicity (Fig. 1c and d; Supplementary Fig. 2e) (Ruaud and Bessereau 2007). Although we observed a slight lengthening of both L1 and L2 by ∼1.1- to 1.2-fold in both *catp-1* alleles, no strong or specific effect was observed at the L2 stage (Fig. 3a and c). As a further assessment, we timed molts using the P*_mlt-10_*::GFP–PEST marker, which peaks in expression for several hours during each molting cycle (Frand *et al*. 2005). Based on this marker, *catp-1(ok2585)* mutants displayed a ∼1.2-fold increase in the length of L1 but exhibited little or no increase in the length of L2 (Fig. 3b and c). We do note, however, that *catp-1(ok2585)* mutants exhibited peaks of P*_mlt-10_*::GFP–PEST expression during each molt that were substantially shorter in duration than wild type, although the meaning of this observation is unclear. Overall, we failed to detect a specific lengthening of the L2 stage, nor were strong effects on stage length observed at L3 or L4 stages based on the P*_mlt-10_*::GFP–PEST marker (Fig. 3b and c).

As a final test for L2d induction, we determined whether an established marker for L2d is inappropriately expressed under normal growth conditions in *catp-1(ok2585)* mutants. P*_ets-10_*::GFP is mostly undetectable in fed wild-type larvae but is expressed robustly after wild-type larvae are treated with dauer-inducing pheromones (Fig. 3d) (Shih *et al*. 2019; Binti *et al*. 2022). We found that *catp-1(ok2585)* larvae did not express the L2d marker under normal growth conditions. Taken together, our data failed to support the model that loss of CATP-1 activity induces L2d, suggesting that suppression of DMPP toxicity and *nekl*-associated molting defects by *catp-1* mutations occurs through another mechanism.

## Discussion

Here, we show that loss of the CATP-1 Na^+^/K^+^ exchanger led to the suppression of molting defects in *nekl-2; nekl-3* mutants. We also show that a mutation designed to specifically abolish CATP-1 ion pump activity was sufficient to cause robust suppression. This is consistent with a recent report implicating CATP-1 pump activity in glia (Johnson *et al*. 2020) but contrasts with data from Ruaud and Bessereau (2007) suggesting pump-independent functions for CATP-1 within the context of DMPP toxicity suppression (Johnson *et al*. 2020). We note that our finding that a CRISPR allele of *catp-1*(D409E) only weakly suppressed DMPP toxicity could reflect CATP-1 pump-independent functions or may be due to partial inactivation of the pump by this mutation. Consistent with findings by Ruaud and Bessereau (2007), we observed expression of CATP-1 throughout the epidermis Moreover, we observed CATP-1 in punctate and reticular structures at or near the apical surface of epidermal cells, suggesting that CATP-1 may function at the plasma membrane and possibly endosomes, broadly consistent with previous studies on P4-type Na^+^/K^+^ pumps (Kaplan 2002; Clausen *et al*. 2017). Collectively, our data indicate that *nekl* molting defects may be partially alleviated by altering the concentration or distribution of intracellular solutes or by changes in electrochemical gradients.

One unexpected finding from our genetic analysis was that presumed strong reduction-of-function alleles of *catp-1* were not effective at suppressing molting defects in *nekl-2; nekl-3* mutants. In contrast, all tested alleles of *catp-1* were at least partially effective at reducing the toxicity of DMPP, with the presumed null allele of *catp-1(ok2585)* appearing to confer the greatest resistance levels. The former observation may be explained in part by a previous report showing that *catp-2*, a paralog of *catp-1*, is transcriptionally upregulated >2,000-fold in *catp-1(kr17)* mutants (Johnson *et al*. 2020). Thus, upregulation of *catp-2* in strong reduction-of-function *catp-1* alleles could potentially compensate for loss of CATP-1 function, thereby preventing suppression. This model predicts that sufficient upregulation of *catp-2* would not occur in alleles of *catp-1* that are capable of suppressing *nekl* defects.

Based on prior studies of *catp-1* and other DMPP toxicity suppressors (Ruaud and Bessereau 2006, 2007; Ruaud *et al*. 2011), we tested the hypothesis that loss of CATP-1 leads to *nekl* suppression by inducing a predauer L2d state, which we recently showed can bypass L2/L3 molting defects in *nekl-2; nekl-3* mutants (Binti *et al*. 2022). Our genetic evidence, however, did not support this model. Nevertheless, we did not assess L2 length in *catp-1* mutants capable of suppressing *nekl* defects, leaving open the possibility that certain alleles of *catp-1* can induce L2d in a manner that is independent of DAF-5. However, we were also unable to recapitulate a previous finding that loss of *catp-1* leads to a marked lengthening of the L2 stage, a hallmark of L2d (Ruaud and Bessereau 2007). Although we have no simple explanation to account for these differences, we note that disparities could potentially arise from altered growth conditions or uncharacterized mutations present in strain backgrounds. It is also worth noting that *catp-1* was suggested to suppress DMPP toxicity through a dauer-independent mechanism (Ruaud and Bessereau 2007). Correspondingly, although the suppression of DMPP toxicity by other dauer-inducing mutations correlated strongly with increased L2 duration, it did not correlate with the ability of these mutants to induce mature dauer larvae (Ruaud and Bessereau 2007; Ruaud *et al*. 2011). Thus, while not fully resolved, our collective data appear to suggest roles for CATP-1 that are independent of the dauer pathway and L2d induction.

WGS of *nekl* suppressor strains identified 3 isolates containing unique mutations that alter the coding region of *catp-1* [WY1286 (*fd170*), WY1768 (*fd168*), and WY1744 (*fd274*)]. Traditionally, the detection of variants at the same locus in 2 or more independent isolates is thought to imply that the common locus is likely responsible for causing the phenotype of interest. This logic is particularly compelling if mutations at that locus have already been shown to be causative and sufficient, as was the case for *catp-1* (WY1286). We therefore anticipated that the mutations impacting the coding region of *catp-1* in strains WY1768 and WY1744 would be responsible for the observed suppression. Surprisingly, CRISPR phenocopy of these *catp-1* variants failed to suppress *nekl-2; nekl-3* molting defects, indicating that they are insufficient for suppression. Nevertheless, it is plausible that these mutations may contribute to the observed suppression and, may even be necessary, in conjunction with other loci. More broadly, these findings stress the importance of taking a cautious approach in cases where a known casual gene is identified as a candidate in other sequenced strains.

It was previously shown that the CATP-1a isoform is sufficient for rescuing both DMPP toxicity (Ruaud and Bessereau 2007) and neuronal sensory phenotypes in *catp-1* mutants (Johnson *et al*. 2020), indicating that CATP-1a may be functionally sufficient in epidermal and glial cells, respectively. Interestingly, the failure of the GT > AT *catp-1a* intron 15 splice-site mutation to suppress *nekl-2; nekl-3* mutants suggests that the CATP-1b isoform may be able to partially compensate for the loss of CATP-1a. We note that an alternative in-frame splice donor sequence (GT) is not present within intronic sequences immediately downstream of the splice-site mutation, and although several upstream GT sequences in exon 15 could potentially serve this role, they would lead to the partial removal of the CATP-1a ninth TM domain. Notably, CATP-1b is not predicted to encode a ninth or tenth TM domain, and functional P4-ATPases have been reported to contain as few as 6 TM domains, with TM1–6 making up the functional core (Kuhlbrandt 2004; Morth *et al*. 2011). Taken together, our data suggest that CATP-1a and CATP-1b may be partially functionally redundant and that both isoforms may encode functional Na^+^/K^+^ pumps.

In prior studies, we have shown that NEKL-2 and NEKL-3 localize to several endosomal trafficking compartments, that depletion of NEKL-2 and NEKL-3 leads to morphologically and functionally altered trafficking compartments, and that mutations in several membrane trafficking factors can suppress molting defects associated with *nekl* loss (Yochem *et al*. 2015; Lazetic and Fay 2017a; Lazetic *et al*. 2018; Joseph *et al*. 2020; Joseph *et al*. 2023). Thus, it is possible that loss of *catp-1* may lead to a partial suppression of *nekl* molting defects through direct or indirect effects on membrane trafficking. Some support for this model comes from published studies linking cation levels and electrochemical gradients to effects on membrane trafficking. For example, depletion of intracellular potassium leads to the inhibition of clathrin-coated pit formation, potentially by preventing clathrin from interacting with its adapter proteins (Larkin *et al*. 1983; Heuser and Anderson 1989; Hansen *et al*. 1993). Correspondingly, we found that inhibition of AP2 clathrin-adapter activation can suppress *nekl* molting and trafficking-associated defects (Joseph *et al*. 2020). Na^+^/K^+^ pumps also affect the acidification and function of early endosomes by impacting the movement of H^+^ across endosomal membranes, and the pharmacological inhibition of Na^+^/K^+^ pumps leads to endosomal recycling defects (Fuchs *et al*. 1989; Rosen *et al*. 2004, 2006; Feldmann *et al*. 2007). Finally, ionic gradients have been shown to affect vesicle morphology and motility through changes in membrane tension and altered cytoskeletal dynamics (Nunes *et al*. 2015; Simunovic and Voth 2015; Murray *et al*. 2017; Mercier *et al*. 2020; Saric and Freeman 2021).

In addition to the above-mentioned effects on membrane trafficking, Na^+^/K^+^ pumps affect various cell signaling pathways, which could represent another means by which *catp-1* loss may impact NEKL functions and molting (Davis *et al*. 1995; Mohammadi *et al*. 2001; Liang *et al*. 2006; Doi and Iwasaki 2008; Matchkov and Krivoi 2016; Clausen *et al*. 2017; Pivovarov *et al*. 2018). Likewise, analogous to its function in glia, CATP-1 could potentially modify the activity of neurons that are closely associated with the epidermis, which might conceivably impact the molting process (Johnson *et al*. 2020). It is also worth noting that intracellular potassium oscillations have been implicated in circadian rhythms in a range of cell types (Feeney *et al*. 2016; Henslee *et al*. 2017; Rodriguez *et al*. 2024) and that connections have been made between genes regulating circadian rhythms and molting in *C. elegans* (Monsalve *et al*. 2011; Monsalve and Frand 2012; Lazetic and Fay 2017b; Ragle *et al*. 2020; Patel *et al*. 2022; Lamberti *et al*. 2024). At present, however, it remains to be determined precisely how *catp-1* and changes in cation concentration can compensate for *nekl* reduction of function. Future studies will examine how mutations in *catp-1* and other identified *nekl* suppressors impact membrane trafficking processes and whether they suppress 1 or more membrane trafficking defects associated with *nekl* loss of function.

## Supplementary Material

jkae244_Supplementary_Data

## Data Availability

Data from all experiments are given in Supplementary File 3. All reagents generated in this study will be made available upon request. The authors affirm that all data necessary for confirming the conclusions of the article are present within the article, figures, and tables. Supplemental material available at G3 online.

## References

[jkae244-B1] Aguinaldo AM , Turbeville JM, Linford LS, Rivera MC, Garey JR, Raff RA, Lake JA. 1997. Evidence for a clade of nematodes, arthropods and other moulting animals. Nature. 387(6632):489–493. doi:10.1038/387489a0. 9168109

[jkae244-B2] Arribere JA , Bell RT, Fu BX, Artiles KL, Hartman PS, Fire AZ. 2014. Efficient marker-free recovery of custom genetic modifications with CRISPR/Cas9 in *Caenorhabditis elegans*. Genetics. 198(3):837–846. doi:10.1534/genetics.114.169730. 25161212 PMC4224173

[jkae244-B3] Asahina M , Ishihara T, Jindra M, Kohara Y, Katsura I, Hirose S. 2000. The conserved nuclear receptor Ftz-F1 is required for embryogenesis, moulting and reproduction in *Caenorhabditis elegans*. Genes Cells. 5(9):711–723. doi:10.1046/j.1365-2443.2000.00361.x. 10971653

[jkae244-B4] Binti S , Melinda RV, Joseph BB, Edeen PT, Miller SD, Fay DS. 2022. A life cycle alteration can correct molting defects in *Caenorhabditis elegans*. Dev Biol. 483:143–156. doi:10.1016/j.ydbio.2022.01.001. 35038442 PMC8867747

[jkae244-B5] C. elegans Deletion Mutant Consortium . 2012. Large-scale screening for targeted knockouts in the *Caenorhabditis elegans* genome. G3 (Bethesda). 2(11):1415–1425. doi:10.1534/g3.112.003830. 23173093 PMC3484672

[jkae244-B6] Clancy JC , Vo AA, Myles KM, Levenson MT, Ragle JM, Ward JD. 2023. Experimental considerations for study of *C. elegans* lysosomal proteins. G3 (Bethesda). 13(4), jkad032. doi:10.1093/g3journal/jkad032. 36748711 PMC10085801

[jkae244-B7] Clausen MV , Hilbers F, Poulsen H. 2017. The structure and function of the Na,K-ATPase isoforms in health and disease. Front Physiol. 8:371. doi:10.3389/fphys.2017.00371. 28634454 PMC5459889

[jkae244-B8] Davis MW , Somerville D, Lee RY, Lockery S, Avery L, Fambrough DM. 1995. Mutations in the Caenorhabditis elegans Na,K-ATPase alpha-subunit gene, *eat-6*, disrupt excitable cell function. J Neurosci. 15(12):8408–8418. doi:10.1523/JNEUROSCI.15-12-08408.1995. 8613772 PMC4445131

[jkae244-B9] Dickinson DJ , Goldstein B. 2016. CRISPR-based methods for *Caenorhabditis elegans* genome engineering. Genetics. 202(3):885–901. doi:10.1534/genetics.115.182162. 26953268 PMC4788126

[jkae244-B10] Dickinson DJ , Pani AM, Heppert JK, Higgins CD, Goldstein B. 2015. Streamlined genome engineering with a self-excising drug selection cassette. Genetics. 200(4):1035–1049. doi:10.1534/genetics.115.178335. 26044593 PMC4574250

[jkae244-B11] Doi M , Iwasaki K. 2008. Na+/K+ ATPase regulates the expression and localization of acetylcholine receptors in a pump activity-independent manner. Mol Cell Neurosci. 38(4):548–558. doi:10.1016/j.mcn.2008.05.003. 18599311 PMC2569892

[jkae244-B12] Entchev EV , Kurzchalia TV. 2005. Requirement of sterols in the life cycle of the nematode *Caenorhabditis elegans*. Semin Cell Dev Biol. 16(2):175–182. doi:10.1016/j.semcdb.2005.01.004. 15797828

[jkae244-B13] Ewer J . 2005. How the ecdysozoan changed its coat. PLoS Biol. 3(10):e349. doi:10.1371/journal.pbio.0030349. 16207077 PMC1250302

[jkae244-B14] Farboud B , Meyer BJ. 2015. Dramatic enhancement of genome editing by CRISPR/Cas9 through improved guide RNA design. Genetics. 199(4):959–971. doi:10.1534/genetics.115.175166. 25695951 PMC4391549

[jkae244-B15] Farboud B , Severson AF, Meyer BJ. 2019. Strategies for efficient genome editing using CRISPR-Cas9. Genetics. 211(2):431–457. doi:10.1534/genetics.118.301775. 30504364 PMC6366907

[jkae244-B16] Farley R . 2012. Active ion transport by ATP-driven ion pumps. In: Sperelakis N, editor. Cell physiology sourcebook. Cambridge, MA: Academic Press/Elsevier Inc.

[jkae244-B17] Fay SF , Fay DS, Chhatre VE. 2021. CRISPRcruncher: a tool for engineering restriction sites into coding regions. MicroPubl Biol. doi:10.17912/micropub.biology.000343. PMC7816087 33490886

[jkae244-B18] Fay DS , Gerow K. 2013. A biologist's guide to statistical thinking and analysis. WormBook. 1–54. doi:10.1895/wormbook.1.159.1. PMC3880567 23908055

[jkae244-B19] Feeney KA , Hansen LL, Putker M, Olivares-Yañez C, Day J, Eades LJ, Larrondo LF, Hoyle NP, O'Neill JS, van Ooijen G. 2016. Daily magnesium fluxes regulate cellular timekeeping and energy balance. Nature. 532(7599):375–379. doi:10.1038/nature17407. 27074515 PMC4886825

[jkae244-B20] Feldmann T , Glukmann V, Medvenev E, Shpolansky U, Galili D, Lichtstein D, Rosen H. 2007. Role of endosomal Na+-K+-ATPase and cardiac steroids in the regulation of endocytosis. Am J Physiol Cell Physiol. 293(3):C885–C896. doi:10.1152/ajpcell.00602.2006. 17553933

[jkae244-B21] Fielenbach N , Antebi A. 2008. *C. elegans* dauer formation and the molecular basis of plasticity. Genes Dev. 22(16):2149–2165. doi:10.1101/gad.1701508. 18708575 PMC2735354

[jkae244-B22] Frand AR , Russel S, Ruvkun G. 2005. Functional genomic analysis of *C. elegans* molting. PLoS Biol. 3(10):e312. doi:10.1371/journal.pbio.0030312. 16122351 PMC1233573

[jkae244-B23] Fuchs R , Schmid S, Mellman I. 1989. A possible role for Na+,K+-ATPase in regulating ATP-dependent endosome acidification. Proc Natl Acad Sci U S A. 86(2):539–543. doi:10.1073/pnas.86.2.539. 2536167 PMC286507

[jkae244-B24] Ghanta KS , Ishidate T, Mello CC. 2021. Microinjection for precision genome editing in *Caenorhabditis elegans*. STAR Protoc. 2(3):100748. doi:10.1016/j.xpro.2021.100748. 34505086 PMC8417391

[jkae244-B25] Gissendanner CR , Sluder AE. 2000. *nhr-25*, the *Caenorhabditis elegans* ortholog of *ftz-f1*, is required for epidermal and somatic gonad development. Dev Biol. 221(1):259–272. doi:10.1006/dbio.2000.9679. 10772806

[jkae244-B26] Golden JW , Riddle DL. 1984. The *Caenorhabditis elegans* dauer larva: developmental effects of pheromone, food, and temperature. Dev Biol. 102(2):368–378. doi:10.1016/0012-1606(84)90201-X. 6706004

[jkae244-B27] Hansen SH , Sandvig K, van Deurs B. 1993. Clathrin and HA2 adaptors: effects of potassium depletion, hypertonic medium, and cytosol acidification. J Cell Biol. 121(1):61–72. doi:10.1083/jcb.121.1.61. 8458873 PMC2119777

[jkae244-B28] Harris TW , Arnaboldi V, Cain S, Chan J, Chen WJ, Cho J, Davis P, Gao S, Grove CA, Kishore R, et al 2020. WormBase: a modern model organism information resource. Nucleic Acids Res. 48(D1):D762–D767. doi:10.1093/nar/gkz920. 31642470 PMC7145598

[jkae244-B29] Henslee EA , Crosby P, Kitcatt SJ, Parry JSW, Bernardini A, Abdallat RG, Braun G, Fatoyinbo HO, Harrison EJ, Edgar RS, et al 2017. Rhythmic potassium transport regulates the circadian clock in human red blood cells. Nat Commun. 8(1):1978. doi:10.1038/s41467-017-02161-4. 29215003 PMC5719349

[jkae244-B30] Heuser JE , Anderson RG. 1989. Hypertonic media inhibit receptor-mediated endocytosis by blocking clathrin-coated pit formation. J Cell Biol. 108(2):389–400. doi:10.1083/jcb.108.2.389. 2563728 PMC2115439

[jkae244-B31] Jacobsen MD , Pedersen PA, Jorgensen PL. 2002. Importance of Na,K-ATPase residue alpha 1-Arg544 in the segment Arg544-Asp567 for high-affinity binding of ATP, ADP, or MgATP. Biochemistry. 41(5):1451–1456. doi:10.1021/bi015891h. 11814337

[jkae244-B32] Johnson CK , Fernandez-Abascal J, Wang Y, Wang L, Bianchi L. 2020. The Na(+)-K(+)-ATPase is needed in glia of touch receptors for responses to touch in *C. elegans*. J Neurophysiol. 123(5):2064–2074. doi:10.1152/jn.00636.2019. 32292107 PMC7444924

[jkae244-B33] Johnson LC , Vo AA, Clancy JC, Myles KM, Pooranachithra M, Aguilera J, Levenson MT, Wohlenberg C, Rechtsteiner A, Ragle JM, et al 2023. NHR-23 activity is necessary for *C. elegans* developmental progression and apical extracellular matrix structure and function. Development. 150(10):dev201085. doi:10.1242/dev.201085. 37129010 PMC10233720

[jkae244-B34] Johnstone IL , Barry JD. 1996. Temporal reiteration of a precise gene expression pattern during nematode development. EMBO J. 15(14):3633–3639. doi:10.1002/j.1460-2075.1996.tb00732.x. 8670866 PMC451985

[jkae244-B35] Joseph BB , Blouin NA, Fay DS. 2018. Use of a sibling subtraction method for identifying causal mutations in *Caenorhabditis elegans* by whole-genome sequencing. G3 (Bethesda). 8(2):669–678. doi:10.1534/g3.117.300135. 29237702 PMC5919755

[jkae244-B36] Joseph BB , Edeen PT, Meadows S, Binti S, Fay DS. 2022. An unexpected role for the conserved ADAM-family metalloprotease ADM-2 in *Caenorhabditis elegans* molting. PLoS Genet. 18(5):e1010249. doi:10.1371/journal.pgen.1010249. 35639786 PMC9187072

[jkae244-B37] Joseph BB , Naslavsky N, Binti S, Conquest S, Robison L, Bai G, Homer RO, Grant BD, Caplan S, Fay DS. 2023. Conserved NIMA kinases regulate multiple steps of endocytic trafficking. PLoS Genet. 19(4):e1010741. doi:10.1371/journal.pgen.1010741. 37099601 PMC10166553

[jkae244-B38] Joseph BB , Wang Y, Edeen P, Lažetić V, Grant BD, Fay DS. 2020. Control of clathrin-mediated endocytosis by NIMA family kinases. PLoS Genet. 16(2):e1008633. doi:10.1371/journal.pgen.1008633. 32069276 PMC7048319

[jkae244-B39] Kang YL , Yochem J, Bell L, Sorensen EB, Chen L, Conner SD. 2013. *Caenorhabditis elegans* reveals a FxNPxY-independent low-density lipoprotein receptor internalization mechanism mediated by epsin1. Mol Biol Cell. 24(3):308–318. doi:10.1091/mbc.e12-02-0163. 23242996 PMC3564534

[jkae244-B40] Kaplan JH . 2002. Biochemistry of Na,K-ATPase. Annu Rev Biochem. 71(1):511–535. doi:10.1146/annurev.biochem.71.102201.141218. 12045105

[jkae244-B41] Karp X . 2018. Working with dauer larvae. WormBook. 2018:1–19. doi:10.1895/wormbook.1.180.1. PMC5237411 27417559

[jkae244-B42] Kim H , Ishidate T, Ghanta KS, Seth M, Conte D Jr, Shirayama M, Mello CC. 2014. A co-CRISPR strategy for efficient genome editing in *Caenorhabditis elegans*. Genetics. 197(4):1069–1080. doi:10.1534/genetics.114.166389. 24879462 PMC4125384

[jkae244-B43] Kostrouchova M , Krause M, Kostrouch Z, Rall JE. 2001. Nuclear hormone receptor CHR3 is a critical regulator of all four larval molts of the nematode *Caenorhabditis elegans*. Proc Natl Acad Sci U S A. 98(13):7360–7365. doi:10.1073/pnas.131171898. 11416209 PMC34673

[jkae244-B44] Kuhlbrandt W . 2004. Biology, structure and mechanism of P-type ATPases. Nat Rev Mol Cell Biol. 5(4):282–295. doi:10.1038/nrm1354. 15071553

[jkae244-B45] Kutscher LM , Shaham S. 2014. Forward and reverse mutagenesis in *C. elegans*. WormBook. 1–26. doi:10.1895/wormbook.1.167.1. PMC4078664 24449699

[jkae244-B46] Lamberti ML , Spangler RK, Cerdeira V, Ares M, Rivollet L, Ashley GE, Coronado AR, Tripathi S, Spiousas I, Ward JD, et al 2024. Clock gene homologs lin-42 and kin-20 regulate circadian rhythms in *C. elegans*. Sci Rep. 14(1):12936. doi:10.1038/s41598-024-62303-9. 38839826 PMC11153552

[jkae244-B47] Larkin JM , Brown MS, Goldstein JL, Anderson RG. 1983. Depletion of intracellular potassium arrests coated pit formation and receptor-mediated endocytosis in fibroblasts. Cell. 33(1):273–285. doi:10.1016/0092-8674(83)90356-2. 6147196

[jkae244-B48] Lazetic V , Fay DS. 2017a. Conserved ankyrin repeat proteins and their NIMA kinase partners regulate extracellular matrix remodeling and intracellular trafficking in *Caenorhabditis elegans*. Genetics. 205(1):273–293. doi:10.1534/genetics.116.194464. 27799278 PMC5223508

[jkae244-B49] Lazetic V , Fay DS. 2017b. Molting in *C. elegans*. Worm. 6(1):e1330246. doi:10.1080/21624054.2017.1330246. 28702275 PMC5501215

[jkae244-B50] Lazetic V , Joseph BB, Bernazzani SM, Fay DS. 2018. Actin organization and endocytic trafficking are controlled by a network linking NIMA-related kinases to the CDC-42-SID-3/ACK1 pathway. PLoS Genet. 14(4):e1007313. doi:10.1371/journal.pgen.1007313. 29608564 PMC5897031

[jkae244-B51] Li JJ , Huang H, Bickel PJ, Brenner SE. 2014. Comparison of *D. melanogaster* and *C. elegans* developmental stages, tissues, and cells by modENCODE RNA-seq data. Genome Res. 24(7):1086–1101. doi:10.1101/gr.170100.113. 24985912 PMC4079965

[jkae244-B52] Liang M , Cai T, Tian J, Qu W, Xie ZJ. 2006. Functional characterization of Src-interacting Na/K-ATPase using RNA interference assay. J Biol Chem. 281(28):19709–19719. doi:10.1074/jbc.M512240200. 16698801

[jkae244-B53] Martin R , Entchev EV, Kurzchalia TV, Knolker HJ. 2010. Steroid hormones controlling the life cycle of the nematode *Caenorhabditis elegans*: stereoselective synthesis and biology. Org Biomol Chem. 8(4):739–750. doi:10.1039/B918488K. 20135027

[jkae244-B54] Matchkov VV , Krivoi II. 2016. Specialized functional diversity and interactions of the Na,K-ATPase. Front Physiol. 7:179. doi:10.3389/fphys.2016.00179. 27252653 PMC4879863

[jkae244-B55] Mercier V , Larios J, Molinard G, Goujon A, Matile S, Gruenberg J, Roux A. 2020. Endosomal membrane tension regulates ESCRT-III-dependent intra-lumenal vesicle formation. Nat Cell Biol. 22(8):947–959. doi:10.1038/s41556-020-0546-4. 32753669 PMC7612185

[jkae244-B56] Merris M , Wadsworth WG, Khamrai U, Bittman R, Chitwood DJ, Lenard J. 2003. Sterol effects and sites of sterol accumulation in *Caenorhabditis elegans*: developmental requirement for 4alpha-methyl sterols. J Lipid Res. 44(1):172–181. doi:10.1194/jlr.M200323-JLR200. 12518036

[jkae244-B57] Miao R , Li M, Zhang Q, Yang C, Wang X. 2020. An ECM-to-nucleus signaling pathway activates lysosomes for *C. elegans* larval development. Dev Cell. 52(1):21–37.e5. doi:10.1016/j.devcel.2019.10.020. 31735670

[jkae244-B58] Mohammadi K , Kometiani P, Xie Z, Askari A. 2001. Role of protein kinase C in the signal pathways that link Na+/K+-ATPase to ERK1/2. J Biol Chem. 276(45):42050–42056. doi:10.1074/jbc.M107892200. 11562372

[jkae244-B59] Monsalve GC , Frand AR. 2012. Toward a unified model of developmental timing: a “molting” approach. Worm. 1(4):221–230. doi:10.4161/worm.20874. 24058853 PMC3670223

[jkae244-B60] Monsalve GC , Van Buskirk C, Frand AR. 2011. LIN-42/PERIOD controls cyclical and developmental progression of *C. elegans* molts. Curr Biol. 21(24):2033–2045. doi:10.1016/j.cub.2011.10.054. 22137474

[jkae244-B61] Morth JP , Pedersen BP, Buch-Pedersen MJ, Andersen JP, Vilsen B, Palmgren MG, Nissen P. 2011. A structural overview of the plasma membrane Na+,K+-ATPase and H+-ATPase ion pumps. Nat Rev Mol Cell Biol. 12(1):60–70. doi:10.1038/nrm3031. 21179061

[jkae244-B62] Murray JW , Yin D, Wolkoff AW. 2017. Reduction of organelle motility by removal of potassium and other solutes. PLoS One. 12(9):e0184898. doi:10.1371/journal.pone.0184898. 28922372 PMC5602639

[jkae244-B63] Nunes P , Roth I, Meda P, Féraille E, Brown D, Hasler U. 2015. Ionic imbalance, in addition to molecular crowding, abates cytoskeletal dynamics and vesicle motility during hypertonic stress. Proc Natl Acad Sci U S A. 112(24):E3104–E3113. doi:10.1073/pnas.1421290112. 26045497 PMC4475958

[jkae244-B64] Ohtsubo M , Noguchi S, Takeda K, Morohashi M, Kawamura M. 1990. Site-directed mutagenesis of Asp-376, the catalytic phosphorylation site, and Lys-507, the putative ATP-binding site, of the alpha-subunit of Torpedo californica Na+/K(+)-ATPase. Biochim Biophys Acta. 1021(2):157–160. doi:10.1016/0005-2736(90)90028-M. 2154258

[jkae244-B65] Page AP , Johnstone IL. 2007. The cuticle. WormBook. 1–15. doi:10.1895/wormbook.1.138.1. PMC4781593 18050497

[jkae244-B66] Paix A , Folkmann A, Rasoloson D, Seydoux G. 2015. High efficiency, homology-directed genome editing in *Caenorhabditis elegans* using CRISPR-Cas9 ribonucleoprotein complexes. Genetics. 201(1):47–54. doi:10.1534/genetics.115.179382. 26187122 PMC4566275

[jkae244-B67] Paix A , Wang Y, Smith HE, Lee CY, Calidas D, Lu T, Smith J, Schmidt H, Krause MW, Seydoux G. 2014. Scalable and versatile genome editing using linear DNAs with microhomology to Cas9 Sites in *Caenorhabditis elegans*. Genetics. 198(4):1347–1356. doi:10.1534/genetics.114.170423. 25249454 PMC4256755

[jkae244-B68] Patel R , Galagali H, Kim JK, Frand AR. 2022. Feedback between a retinoid-related nuclear receptor and the let-7 microRNAs controls the pace and number of molting cycles in *C. elegans*. Elife. 11:e80010. doi:10.7554/eLife.80010. 35968765 PMC9377799

[jkae244-B69] Pivovarov AS , Calahorro F, Walker RJ. 2018. Na(+)/K(+)-pump and neurotransmitter membrane receptors. Invert Neurosci. 19(1):1. doi:10.1007/s10158-018-0221-7. 30488358 PMC6267510

[jkae244-B70] Porta-de-la-Riva M , Fontrodona L, Villanueva A, Ceron J. 2012. Basic *Caenorhabditis elegans* methods: synchronization and observation. J Vis Exp. 64:e4019. doi:10.3791/4019 PMC3607348 22710399

[jkae244-B71] Post RL , Kume S. 1973. Evidence for an aspartyl phosphate residue at the active site of sodium and potassium ion transport adenosine triphosphatase. J Biol Chem. 248(20):6993–7000. doi:10.1016/S0021-9258(19)43350-4. 4270326

[jkae244-B72] Ragle JM , Aita AL, Morrison KN, Martinez-Mendez R, Saeger HN, Ashley GA, Johnson LC, Schubert KA, Shakes DC, Ward JD. 2020. The conserved molting/circadian rhythm regulator NHR-23/NR1F1 serves as an essential co-regulator of *C. elegans* spermatogenesis. Development. 147:dev193862. doi:10.1242/dev.193862. 33060131 PMC7710015

[jkae244-B73] Riddle DL , Swanson MM, Albert PS. 1981. Interacting genes in nematode dauer larva formation. Nature. 290(5808):668–671. doi:10.1038/290668a0. 7219552

[jkae244-B74] Rodríguez SG , Crosby P, Hansen LL, Grünewald E, Beale AD, Spangler RK, Rabbitts BM, Partch CL, Stangherlin A, O’Neill JS et al, 2024 Potassium rhythms couple the circadian clock to the cell cycle [preprint]. bioRxiv 587153. 10.1101/2024.04.02.587153.

[jkae244-B75] Rosen H , Glukhman V, Feldmann T, Fridman E, Lichtstein D. 2004. Cardiac steroids induce changes in recycling of the plasma membrane in human NT2 cells. Mol Biol Cell. 15(3):1044–1054. doi:10.1091/mbc.e03-06-0391. 14718569 PMC363072

[jkae244-B76] Rosen H , Glukmann V, Feldmann T, Fridman E, Lichtstein D. 2006. Short-term effects of cardiac steroids on intracellular membrane traffic in neuronal NT2 cells. Cell Mol Biol (Noisy-le-grand). 52:78–86. doi:10.1170/T764. 17535740

[jkae244-B77] Ruaud AF , Bessereau JL. 2006. Activation of nicotinic receptors uncouples a developmental timer from the molting timer in *C. elegans*. Development. 133(11):2211–2222. doi:10.1242/dev.02392. 16672334

[jkae244-B78] Ruaud AF , Bessereau JL. 2007. The P-type ATPase CATP-1 is a novel regulator of *C. elegans* developmental timing that acts independently of its predicted pump function. Development. 134(5):867–879. doi:10.1242/dev.02790. 17251264

[jkae244-B79] Ruaud AF , Katic I, Bessereau JL. 2011. Insulin/insulin-like growth factor signaling controls non-dauer developmental speed in the nematode *Caenorhabditis elegans*. Genetics. 187(1):337–343. doi:10.1534/genetics.110.123323. 20944013 PMC3018321

[jkae244-B80] Saric A , Freeman SA. 2021. Solutes as controllers of endomembrane dynamics. Nat Rev Mol Cell Biol. 22(4):237–238. doi:10.1038/s41580-021-00334-0. 33479521 PMC7818714

[jkae244-B81] Schindelin J , Arganda-Carreras I, Frise E, Kaynig V, Longair M, Pietzsch T, Preibisch S, Rueden C, Saalfeld S, Schmid B, et al 2012. Fiji: an open-source platform for biological-image analysis. Nat Methods. 9(7):676–682. doi:10.1038/nmeth.2019. 22743772 PMC3855844

[jkae244-B82] Shih PY , Lee JS, Sternberg PW. 2019. Genetic markers enable the verification and manipulation of the dauer entry decision. Dev Biol. 454(2):170–180. doi:10.1016/j.ydbio.2019.06.009. 31242447

[jkae244-B83] Simunovic M , Voth GA. 2015. Membrane tension controls the assembly of curvature-generating proteins. Nat Commun. 6(1):7219. doi:10.1038/ncomms8219. 26008710 PMC4455092

[jkae244-B84] Singh RN , Sulston JE. 1978. Some observations on the moulting of *Caenorhabditis elegans*. Nematologica. 24(1):63–71. doi:10.1163/187529278X00074.

[jkae244-B85] Sternberg PW , Van Auken K, Wang Q, Wright A, Yook K, Zarowiecki M, Arnaboldi V, Becerra A, Brown S, Cain S, et al 2024. WormBase 2024: status and transitioning to Alliance infrastructure. Genetics. 227(1):iyae050. doi:10.1093/genetics/iyae050. 38573366 PMC11075546

[jkae244-B86] Stiernagle T . 2006. Maintenance of *C. elegans*. *C. elegans*. 1–11. doi:10.1895/wormbook.1.101.1. PMC4781397 18050451

[jkae244-B87] Sundaram MV , Pujol N. 2024. The *Caenorhabditis elegans* cuticle and precuticle: a model for studying dynamic apical extracellular matrices in vivo. Genetics. 227(4):iyae072. doi:10.1093/genetics/iyae072. 38995735 PMC11304992

[jkae244-B88] Telford MJ , Bourlat SJ, Economou A, Papillon D, Rota-Stabelli O. 2008. The evolution of the ecdysozoa. Philos Trans R Soc Lond B Biol Sci. 363(1496):1529–1537. doi:10.1098/rstb.2007.2243. 18192181 PMC2614232

[jkae244-B89] Tsiairis C , Grosshans H. 2021. Gene expression oscillations in *C. elegans* underlie a new developmental clock. Curr Top Dev Biol. 144:19–43. doi:10.1016/bs.ctdb.2020.11.001. 33992153

[jkae244-B90] Yochem J , Lazetic V, Bell L, Chen L, Fay D. 2015. *C. elegans* NIMA-related kinases NEKL-2 and NEKL-3 are required for the completion of molting. Dev Biol. 398(2):255–266. doi:10.1016/j.ydbio.2014.12.008. 25523392 PMC4314388

[jkae244-B91] Yochem J , Tuck S, Greenwald I, Han M. 1999. A gp330/megalin-related protein is required in the major epidermis of *Caenorhabditis elegans* for completion of molting. Development. 126(3):597–606. doi:10.1242/dev.126.3.597. 9876188

