# Supplementary figures and images for: Loss of the Na+/K+ cation pump CATP-1 suppresses nekl-associated molting defects
Source: G3 (Bethesda). 2024 Oct 21;14(12):jkae244. doi: 10.1093/g3journal/jkae244 (PMC11631496; doi:10.1093/g3journal/jkae244)

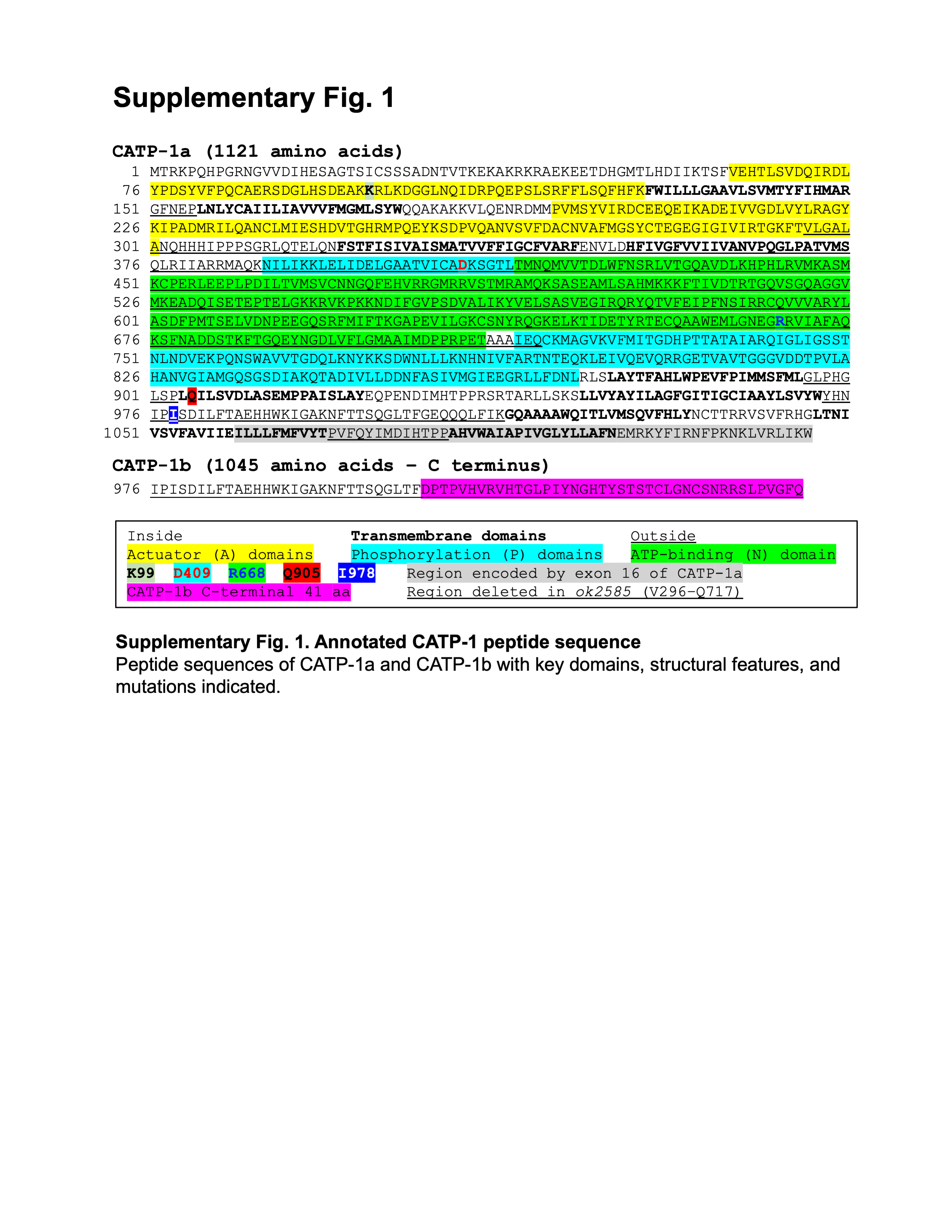

Supplement: jkae244_Supplementary_Data [file jkae244_supplementary_data.zip › Supplementary_Figure_1_G3-2024-405398.png]

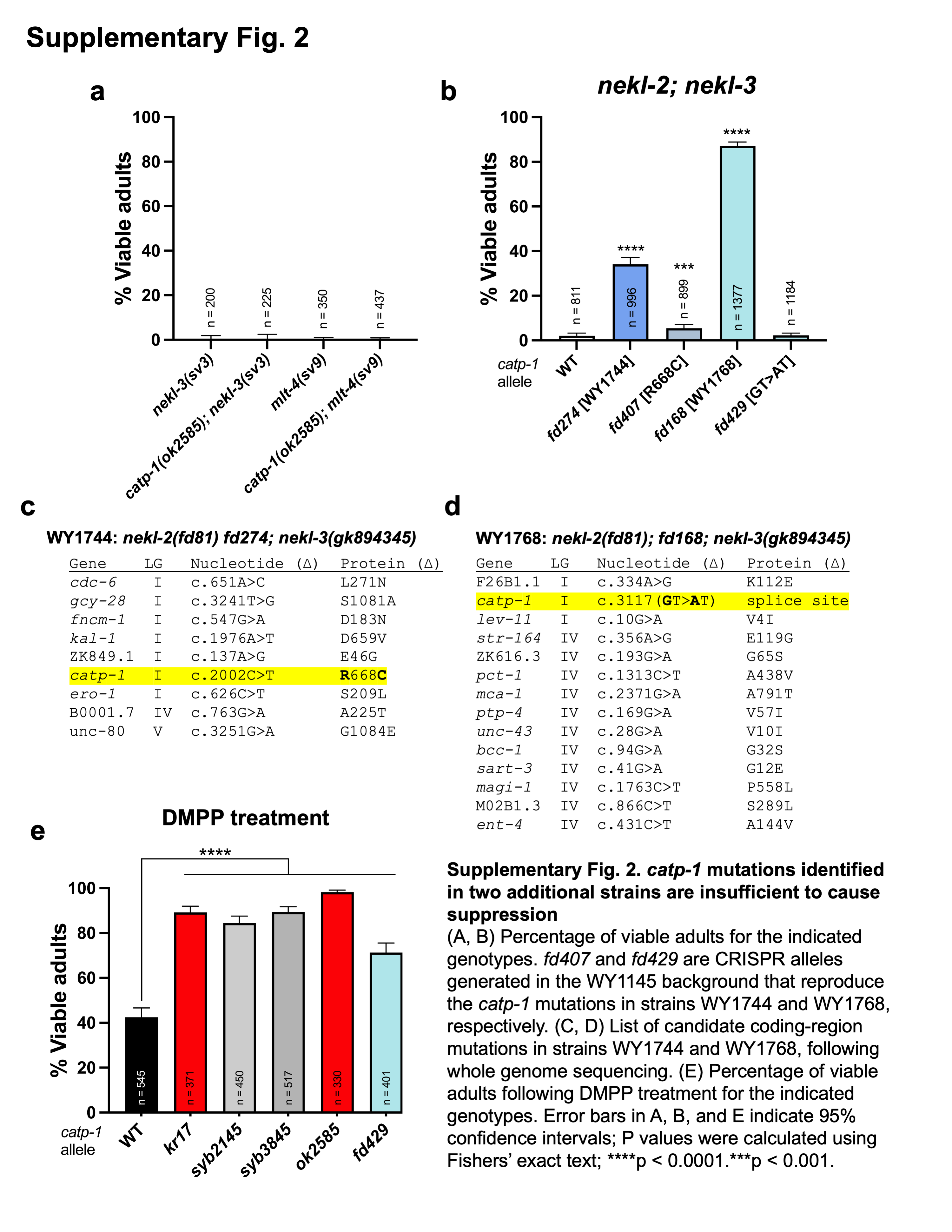

Supplement: jkae244_Supplementary_Data [file jkae244_supplementary_data.zip › Supplementary_Figure_2_G3-2024-405398.png]
